# Carbon dioxide removal from triethanolamine solution using living microalgae-loofah biocomposites

**DOI:** 10.1038/s41598-025-90855-x

**Published:** 2025-02-28

**Authors:** Tanakit Komkhum, Teerawat Sema, Zia Ur Rehman, Pichaya In-na

**Affiliations:** 1 Department of Chemical Technology, Faculty of Science, Chulalongkorn University, Pathumwan, Bangkok, 10330 Thailand; 2 Center of Excellence in Catalysis for Bioenergy and Renewable Chemicals (CBRC), Faculty of Science, Chulalongkorn University, Pathumwan, Bangkok, 10330 Thailand

**Keywords:** Biotechnology, Chemical engineering

## Abstract

Nowadays, the climate change crisis is an urgent matter in which carbon dioxide (CO_2_) is a major greenhouse gas contributing to global warming. Amine solvents are commonly used for CO_2_ capture with high efficiency and absorption rates. However, solvent regeneration consumes an extensive amount of energy. One of alternative approaches is amine regeneration through microalgae. Recently, living biocomposites, intensifying traditional suspended cultivation, have been developed. With this technology, immobilizing microalgae on biocompatible materials with binder outperformed the suspended system in terms of CO_2_ capture rates. In this study, living microalgae-loofah biocomposites with immobilized *Scenedesmus acuminatus* TISTR 8457 using 5%v/v acrylic medium were tested to remove CO_2_ from CO_2_-rich triethanolamine (TEA) solutions. The test using 1 M TEA at various CO_2_ loading ratios (0.2, 0.4, 0.6, and 0.8 mol CO_2_/mol TEA) demonstrated that the biocomposites achieved CO_2_ removal rates 3 to 5 times higher than the suspended cell system over 28 days, with the highest removal observed at the 1 M with 0.4 mol CO_2_/mol TEA (4.34 ± 0.20 g_CO2_/g_biomass_). This study triggers a new exploration of integration between biological and chemical processes that could elevate the traditional amine-based CO_2_ capture capabilities. Nevertheless, pilot-scale investigations are necessary to confirm the biocomposites’s efficiency.

## Introduction

Currently, the climate change situation presents a significant threat to both human societies and natural ecosystems. The ongoing increase in atmospheric carbon dioxide (CO_2_) level, driven primarily by anthropogenic activities such as fossil fuel combustion, deforestation, and industrial processes, is a primary cause of global warming. IPCC (2023) indicated that global CO_2_ emissions surpassed 36.8 Gt/yr^1^, leading to atmospheric CO_2 _concentration exceeding 420 ppm^1^, substantially higher than pre-industrial levels of approximately 280 ppm. This escalation in greenhouse gases has led to numerous adverse environmental impacts, including more frequent and severe weather events, rising sea level, and biodiversity loss^2^. Due to this problem, several research studies have been ongoing to investigate both natural and engineered approaches for addressing this issue.

Afforestation is one of the strategies for controlling CO_2 _concentration by utilizing natural enzymes in photosynthesis process^2^. However, due to a low CO_2_ capture rate of these organisms, the CO_2 _removal by agricultural plants is account for only 3–6% of total fossil fuel emissions^3^. Afforestation for production of bioenergy with carbon capture and storage (BECCS) requires up to 25–75% of global cropland area to achieve the 1.5 °C Paris Agreement target^4^, which negatively affected biodiversity, food and water security, and human existence^2^. To address the land area issue, considerable attention has been directed towards improving CO_2_ removal efficiency using microalgae, as it is known to be an intensified alternative than planting trees. There are many researchers who have been interested in microalgal process, accounting the number of publications on microalgae-based CO_2 _sequestration has steadily grown, exceeding 4,000 articles^5^.

Microalgae, unicellular eukaryotic organisms, can be a solution to eliminate the extensive land use^6^ with their potential to capture CO_2 _10–50 times higher than terrestrial plants through photosynthesis by utilizing sunlight, nutrients, and carbon sources^7^. Moreover, the microalgae process is known for an extra benefit to produce value-added products from its biomass, such as biofuels, cosmetics, nutritional supplements, agricultural products, and pharmaceutical products^8^. Nevertheless, the current CO_2_ sequestration situation of microalgae still faces an enormous quantity of CO_2 _emitted from numerous sources. Although there are various efforts to enhance microalgae assimilation through genetic engineering or improvements of cultivation technologies (both closed and open systems), challenges persist, particularly concerning costs associated with downstream biorefinery processes^5^. Consequently, it is essential to investigate and develop additional technologies, integrating engineering knowledge with natural methods, to effectively handle the raising CO_2_ level.

Over the past decades, Carbon Capture, Utilization, and Storage (CCUS) technologies have been developed to tackle CO_2_ emissions. Among them, an amine-based absorption is a prominent method for CO_2 _post-combustion capture from industrial flue gases and other point sources^9^. This technology demonstrates a high CO_2 _capture rate and efficiency compared to alternative methods (e.g. adsorption, membrane, and cryogenic processes)^5,9^. Furthermore, it can also be integrated with existing infrastructure, facilitating their adoption as a viable mitigation strategy in carbon management frameworks^10^. Despite these advantages, the amine-based CO_2 _capture still faces significant challenges, particularly on energy-intensive solvent regeneration, leading to substantial heat input and high operational cost^10,11^. Over 57–70% of the total energy requirement in the amine-based CO_2 _capture process accounts for the solvent regeneration^12^. Additionally, solvent degradation and corrosion issues can affect system performance and longevity^13^.

With the mentioned issues, this research explores an alternative method for amine regeneration by integrating a biological microalgae process, eliminating the limitation of amine-based absorption and microalgae^5,14,15^. The amine can evade the CO_2_ loss to the atmosphere during the cultivation and the dissolved CO_2_ can be disposed by photosynthesis process in microalgae cells using bicarbonate (HCO_3_^−^) as a carbon source, eradicating energy for regeneration of the amine solution. After that, the amine solution can be recycled for further CO_2 _capture within absorption unit^14,15^. However, the high alkalinity and the corrosive of the amine solution may be toxic to the cells. Hence, combining both processes could elevate CO_2 _capture capability if the level of amine solvents usage is acceptable for microalgae growth^14^. In the last decade, there are several studies, investigating effect of amines in absorption process to CO_2_ fixation of microalgae, consisting MEA-*Chlorella *sp.^16,17^, TEA-*Chlorella *sp.^18^, MEA-*Scenedesmus *sp.^19,20^, DEA-*Scenedesmus *sp.^19^, AMP-*Scenedesmus *sp.^19^, TEA-*Scenedesmus *sp.^19^, DEA-*Spirulina *sp.^21^, MEA-*Spirulina *sp.^22^, TETA-*Scenedesmus *sp.^23^, TMEDA-*Chlorella *sp.^15^, DACH-*Chlorella *sp.^15^, and TEA-*Coccomyxa *sp.^24^.

Most research focused on integrating amines with microalgae in a single system, where amines act as catalysts to capture CO_2_ in solution before it is utilized by the microalgae. They directly contained the amines to culture medium, followed by bubbling CO_2 _to the microalgae. In 2013, Kim et al. (2013)^19^ investigated the cultivation of *Scenedesmus acuminatus* with various amines (MEA, DEA, AMP, and TEA) in suspended system. *S. acuminatus* is widely utilized in research focused on CO_2_ capture from flue gas due to its rapid growth rate and remarkable tolerance to CO_2_ concentrations up to 70%^25^. This species also demonstrated a high potential for biomass production and carbon fixation, utilizing HCO_3_^−^ as a carbon source^25^. Additionally, it accumulates lipids in greater quantities than other species, making it particularly suitable to produce value-added products such as biofuels^19^. The research conducted by Kim et al. (2013) showed that *S. acuminatus* outperformed other microalgal species in removing dissolved CO_2 _from amine-based solutions under similar amine types^16,17,19–22^. The *S. acuminatus* could coexist with TEA solution with less toxicity. Primary amine (MEA) and secondary amine (DEA) react with CO_2 _to form relatively stable carbamate^19^, which cannot be used as a carbon source for microalgae. On the other hand, tertiary amine (TEA) reacts with water and CO_2_ to form HCO_3_^−^ and protonated TEA (TEAH^+^)^18,19^. Additionally, AMP is a sterically hindered amine with unstable carbamate, allowing it to hydrolyze into HCO_3_^− ^as well^26^. HCO_3_^−^ can be carbon nutrient, carrying into microalgae cells through carbon concentrating mechanism (CCM) and converted into CO_2 _for photosynthesis^15,18^. Due to these mechanisms, the tertiary amine or AMP may be suitable alternatives for amine-based absorption combined with microalgae cultivation process.

For recent studies, Yin et al. (2023)^15^ investigated suspended cultivation of *Chlorella* sp. with 1–15 mM of TMEDA and DACH. It was found that TMEDA inhibited cell growth at higher than 1 mM, while DACH at 1–15 mM remained biomass concentration equivalent to the control without inhibitory effects. Regarding pH stability, the use of these amines mitigated pH fluctuations compared to the control. The diurnal increase in pH also indicated active metabolic processes in the microalgae. *Chlorella *sp. demonstrated superior carbon storage and conversion efficiency when cultured with DACH at all tested concentrations, outperforming the control, but TMEDA enhanced carbon conversion efficiency was observed only at 1 mM, with no improvement at higher concentrations^15^. Li et al. (2023)^24^ investigated the effects of induced TEA into *Coccomyxa subellipsoidea* cultures using batch and fed-batch systems. The results showed that adding TEA at a concentration of 100 mg/L enhanced CO_2_ bio-fixation by 1.28-fold and 1.97-fold in the batch and fed-batch systems, respectively, compared to cultures without TEA supplementation. Furthermore, TEA contributed to stabilizing the pH of the culture system and facilitated the conversion of CO_2_ into HCO_3_^⁻^. It also promoted lipid accumulation within the microalgal cells by alleviating oxidative stress, enhancing lipid biosynthesis via glycolysis and the tricarboxylic acid (TCA) cycle. TEA functioned synergistically with *C. subellipsoidea* to capture and utilize CO_2 _more effectively than without TEA or solely microalgae-based cultivation^24^.

Previous studies focused on combining microalgae and amines within the same unit. In contrast, this research emphasizes the removal of CO_2_ from amines that have already been used for CO_2 _capture, which a few research was investigated this approach. Sen and Gurol (2021)^18^ examined separation of amine and microalgae system, initially saturating the TEA solution with CO_2_ prior fed to suspended microalgae without aeration. Interestingly, microalgae demonstrated tolerance to TEA solution, achieving optimal CO_2_ removal at 0.03 M, exceeding CO_2_ removal by more than 2.5 times compared to without TEA. This observation suggested that pre-loading CO_2_ to microalgae culture significantly improved cell viability, as opposed to simultaneous integration of CO_2 _and microalgae in a single system^18^. This finding represented a substantial advancement in enhancing the regeneration potential of TEA via microalgae, facilitated by CO_2_ pre-loading. However, in standalone absorption systems, achieving substantial CO_2_ capture necessitates higher amine concentrations to ensure adequate amine groups are available. Consequently, identifying microalgae strains capable of tolerating high-concentration and enhancing CO_2_ removal from the amine solution to expedite regeneration are critical factors for the effective integration of these dual processes.

Currently, living biocomposites technology, cell immobilization within a coating matrix with non-toxic binders for microbial cell entrapment, which allow the cells to adhere to solid materials, has been developed in various fields such as CO_2 _capture^27–31 ^and wastewater treatment^32,33^. The living microalgae-loofah biocomposites could intensify traditional suspended algae cultivation systems while requiring less space and water. This promising technique also mitigated contamination issues from external substances^34^. To increase long-term durability, immobilization method, binder selection, and support materials are crucial factors for producing effective biocomposites. The materials for cell immobilization should have large surface area for enough cell adhesion, good light penetration to support photosynthesis, non-toxicity to ensure cell viability, good mechanical properties for long-term use, no adverse effects on biological efficiency, support for mass transfer of gases and nutrients, and cost-effectiveness with an ease of scale-up^35,36^. An effective binder must be non-toxic to cells, transparent after drying to allow sufficient light penetration, possess high adhesion capability, and be hydrophilic to facilitate nutrient delivery to the microalgal cells^31,34^. In-na and the team developed biocomposites by immobilizing various microalgae strains, using synthetic binders such as acrylic, styrene, and polyurethane on natural loofah for CO_2_ capture. It was found that acrylic and polyurethane binders were more effective in coexisting with microalgae and facilitating cell immobilization for CO_2 _capture, achieving 9 times higher in the semi-batch system and 18 times higher in the continuous system compared to the suspended system^31^. This finding suggests the potentially increasing the CO_2_ removal from CO_2_-rich TEA solution by microalgae. In addition, the binder can protect the cells and enable the microalgae to withstand higher concentrations of TEA solution.

From the mentioned above, many researchers performed low amine concentrations within suspended system to assist carbon storage in the solution before microalgae utilized CO_2_ as carbon nutrient. In contrast, there was a little research focused on removing rich-CO_2_ amine that absorbed CO_2_ in the absorption unit before by microalgae process, exploring natural ways to recycle amine for further CO_2_ capture, thereby reducing the thermal energy required in conventional CO_2_ desorption processes. However, industrial CO_2_ capture uses high concentration of amine solutions, which can be detrimental on microalgae. To address this limitation, this study aims to apply advanced microalgae process to develop living biocomposites capable of withstanding high concentrations of TEA and to evaluate quantity of CO_2_ removal from CO_2_-rich TEA solutions compared to suspended system. The biocomposites were produced by immobilizing *Scenedesmus acuminatus* TISTR 8457 within an acrylic medium binder on natural loofah scaffold. It is anticipated that, unlike suspended systems, the binder can protect the microalgae from direct exposure to high-concentration TEA, enhancing their tolerance to the high alkalinity environment, and achieving higher and more prolonged CO_2_ removal efficiency. The developed living biocomposites are believed to contribute to Sustainable Development Goals (SDGs), particularly, no.13 Climate Action and no.9 Industry, Innovation, and Infrastructure. This is because they could potentially reduce the overall energy required of the traditional carbon capture unit with an innovative approach.

## Materials and methods

**Fig. 1:**
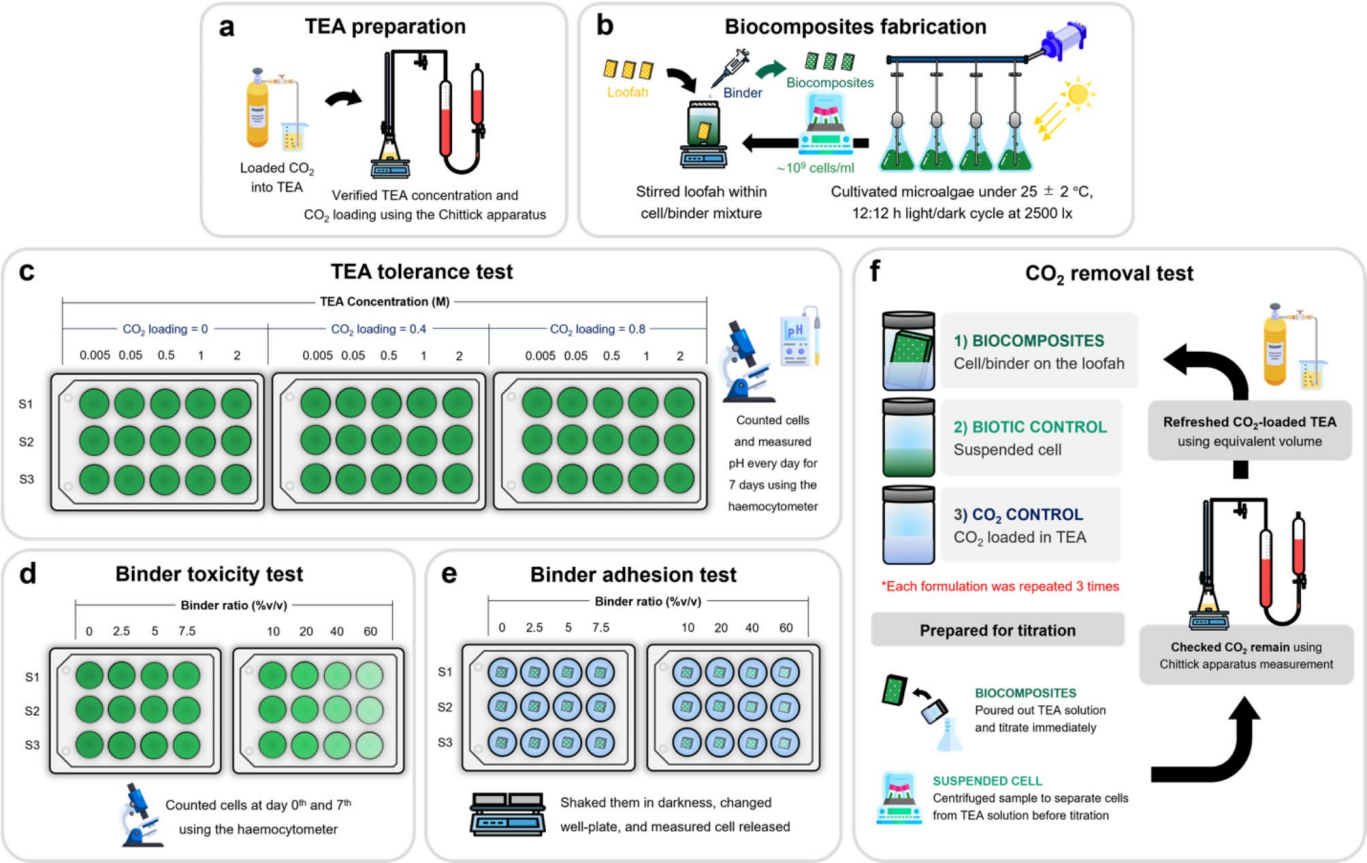
Overview of experimental procedure: (**a**) TEA preparation, (**b**) biocomposites fabrication, (**c**) TEA tolerance test, (**d**) binder toxicity test, (**e**) binder adhesion test, and (**f**) CO_2_ removal test.

An overview of experimental procedure is illustrated in Fig. 1. First, TEA solutions were prepared (Fig. 1a) for testing the tolerance of microalgae to TEA at varying concentrations and CO_2_ loadings (Fig. 1c). Next, biocomposite fabrication was performed (Fig. 1b) to explore the toxicity (Fig. 1d) and adhesion ability (Fig. 1e) of the binder. Finally, the CO_2_ removal from the rich-CO_2_ TEA solution of the biocomposites system was tested by comparing the amount of CO_2_ removal with the suspended system (Fig. 1f).

### Microalgae strain, medium, and growth conditions

The green microalga *Scenedesmus acuminatus *TISTR 8457 was purchased from the Thailand Institute of Scientific and Technological Research (TISTR), Pathum Thani, Thailand. The cells were cultivated in BG-11 medium^37^, performed in 1 L Erlenmeyer flasks with 0.1 vvm of air using aeration pump (RESUN LP-100), and maintained growth conditions at 25 ± 2 °C with a 12:12 h light:dark cycle with 2,500 lx of illumination by LED panel (Fig. 1b).

### TEA tolerance test

TEA (> 99% purity), laboratory grade (KEMAUS), was prepared at various concentrations (0, 0.005, 0.05, 0.5, 1, and 2 M) using BG-11 medium as the diluent (Fig. 1a). Each TEA concentration was supplied with CO_2_ loading levels at 0, 0.4, and 0.8 mol CO_2_/mol TEA, respectively. The TEA concentration and amount of CO_2 _were measured using Chittick apparatus measurement^38^. The CO_2_ loading (Eq. 1) was calculated:

1 $$\:CL\:=\:\frac{{P{V}_{{CO}_{2}}}}{{RT}{C}_{HCl}{\:V}_{HCl}}$$

where $\:CL$ = CO_2_ loading (mol CO_2_/mol TEA), $\:P$ = pressure (kPa), $\:{V}_{{CO}_{2}}$ = volume (L), $\:R$ = ideal gas constant (8.314 (L⋅kPa)/(K⋅mol)), $\:T$ = temperature (K), $\:{C}_{HCl}$ = concentration of HCl (mol/L), $\:{V}_{HCl}$ = volume of HCl (L). After the TEA preparation, the microalgae cells were centrifuged (4,000 rpm for 10 min), poured supernatant, and combined cells to achieve a cell density of approximately 10^9^ cells/mL. The cell density (N) (Eq. 2) was verified using a haemocytometer and calculated, where $\:N$ = cell density (cells/mL).

2 $$\:N=\frac{Number\:of\:cells\:\times\:Dilution\:factor}{Area\:counted\:\times\:Depth\:of\:chamber}$$

The microalgae cells and CO_2_-loaded TEA solution were pipetted into a multi well-plate at a ratio of 1:30 and cultured for 7 days under the same conditions as the microalgae cultivation (25 ± 2 °C, 12:12 h light:dark cycle with LED light at 2,500 lx). Each condition was replicated three times (*n* = 3). The samples were taken daily to count the number of cells and calculate the cell density (Eq. 2) and measured pH daily by Mettler-Toledo AG 8603 pH meter.

### Binder toxicity and binder adhesion tests

These experiments were modified by In-na and the team^29,31^. Acrylic medium (Amsterdam Medium Gloss No. 012) was used as binder for biocomposites fabrication (Fig. 1d and e). The cells ($\:\sim$10^9^ cells/mL) were then mixed with the binder at various ratios (2.5, 5, 7.5, 10, 20, 40, and 60%v/v, respectively). For binder toxicity test, the cells/binder mixture and BG-11 medium were pipetted into a multi well-plate at a ratio of 1:30. The biotic control was microalgae cells without the binder (0%v/v). The samples were cultured for 7 days under the same condition as the microalgae cultivation (25 ± 2 °C, 12:12 h light:dark cycle with LED light at 2500 lx). Each condition was replicated three times (*n* = 3). The samples were photographed daily. On day 0^th^ and 7^th^, the samples were taken daily to count the number of cells and calculate the cell density (Eq. 2). The specific growth rate (Eq. 3) was calculated using cell density between day 0^th^ and 7^th^, where $\:\dot{N}$= specific growth rate (d^[-2^), $\:t$ = time (d).

3 $$\:\dot{N}=\frac{ln\left({N}_{day\:7}/{N}_{day\:0}\right)}{{t}_{day\:7}-{t}_{day\:0}}$$

For binder adhesion test, natural loofah (purchased from local market, Udon Thani, Thailand) was cut into approximately 1 × 1 × 1 cm^3^, autoclaved, and then dried in an oven at 105 °C for 3 h. The loofah samples were submerged in the cells/binder mixture for each binder ratio for 2 min with a magnetic bar to ensure uniform mixing of the microalgae cells and acrylic medium. The biotic control used microalgae cells without the binder. Each condition was replicated three times (*n* = 3). Then, the biocomposites (cells/binder mixture that adhered on the loofah) were dried in an oven at 30 ± 2 °C for 2 h. Afterward, the biocomposites were placed in a multi-well plate and 3 mL of BG-11 medium was added to each well. The multi-well plates were covered and wrapped with aluminum foil. The samples were shaken on an orbital shaker at 80 rpm for 1, 24, 48, and 72 h. The medium was refreshed, and the cells released were counted at each specified time interval to determine the cumulative percentage of cells released from the loofah.

The results were calculated scores for selecting the appropriate binder ratio using a decision matrix^39^. The weighting importance of the cell viability (binder toxicity test) to the adhesive capability (binder adhesion test) was weighted at 3:2^29,31^. The cell viability score (Eq. 4) (maximum 60 points) was calculated using specific growth rate of the binder condition normalized relative to the biotic control, multiplying with weighting of 0.6, where S_1_ = cell viability score.

4 $$\:{S}_{1}=\:\left(\frac{{\dot{N}}_{i\%\:binder}}{{\dot{N}}_{no\:binder}}\times\:100\right)\times\:0.6$$

The adhesive capability score (Eq. 5) (maximum 40 points) was calculated using the percentage of cells released from the loofah, subtracting it from 100%, and normalizing the result where S_2_ = cell adhesion score.

5 $$\:{S}_{2}\:=\:\left[100\:-\:\left(\frac{\%\:cell\:released\:\left(i\%\:binder\right)}{100}\times\:100\right)\right]\times\:0.4$$

Then, the total score (Eq. 6) (maximum 100 points) was obtained from the integration of cell viability and cell adhesion scores.

6 $$\:Total\:score={S}_{1}+{S}_{2}$$

Finally, the total score of each condition was ranked, selecting the highest score as the optimal binder concentration and used to produce biocomposites for further CO_2_ removal test.

The biocomposites, both before and after the binder adhesion test, were observed for their morphology using scanning electron microscopy (SEM) at a low vacuum mode at a voltage of 20 kV. The samples were prepared with dimensions of 0.8 × 0.8 cm^1^ and coated with gold.

### Carbon dioxide removal test

Natural loofah was cut into approximately 3 × 5 × 1 cm^3^, autoclaved, and dried in an oven at 105 °C for 3 h (Fig. 1f). TEA solution was prepared at various concentrations (0.1, 0.25, 0.5, and 1 M, respectively) using BG-11 medium as the diluent. Each TEA concentration was supplied CO_2_ at a ratio of 0.8 mol CO_2_/mol TEA. The experiment was divided into three systems: (1) Biocomposites system, the loofah was submerged in cell/binder mixture for 2 min in a beaker with a magnetic stirrer and dried in an oven at 30 ± 2 °C for 2 h; (2) Suspended system, this system used cell density same as those fixed on the biocomposites ($\:\sim$10^9^ cells/mL and the mass of the cells on biocomposites was weighed to determine the volume required for the suspended system); (3) CO_2_ control system, this system was designed to observe CO_2_ loss from the TEA solution to the atmosphere, consisting only of CO_2_-loaded TEA solution. All three systems were tested in glass bottles containing 30 mL of CO_2_-loaded TEA solution. The bottles were sealed with plastic caps and wrapped with parafilm to prevent accidental CO_2_ leakage. All the samples were cultured for 28 days with the same conditions as the microalgae cultivation (25 ± 2 °C, 12:12 h light:dark cycle with LED light at 2,500 lx). Every four days, the TEA solution from each sample was collected and titrated using the Chittick apparatus to measure the CO_2_ content, observing the amount of CO_2_ removed. For the biocomposites system, the TEA solution was immediately poured out. For the suspended system, the microalgae were centrifuged and supernatant was poured out to separate them from the TEA solution. The TEA solution was refreshed into the bottle with the same concentration and CO_2_ loading. Finally, the cumulative CO_2_ removal (Eq. 7) was calculated, where $\:{m}_{{CO}_{2}}$ = amount of CO_2_ removal (g_CO2_/g_biomass_), $\:{M}_{w}$ = molecular mass of CO_2_ (g/mol), $\:{m}_{b}$ = weight of initial cell biomass (g).

7 $$\:{m}_{{CO}_{2}}=\:\frac{{{PM}_{w}}\left({V}_{{CO}_{2}}\:control\:-\:{V}_{{CO}_{2}}\:sample\right)}{{RT}{m}_{b}}$$

After completing the test with different TEA concentration conditions, the CO_2_ removal from the TEA solution with different CO_2_ loading using 1 M TEA solution (CO_2_ loading levels of 0.2, 0.4, 0.6, and 0.8 mol CO_2_/mol TEA, respectively) was studied. This experiment was similar to the previous test, involving biocomposites system, suspended system, and CO_2_ control system. The samples were cultured for 28 days under the same conditions as the microalgae cultivation (25 ± 2 °C, 12:12 h light:dark cycle with LED light at 2,500 lx). Each condition was replicated three times (*n* = 3).

### Statistical analysis

Microsoft Excel with Real Statistics Add-in was used for statistical data analysis. All experimental results were presented as mean ± standard deviation. The normality of the data distribution was tested using the Shapiro-Wilk Test. For normally distributed data, ANOVA followed by Tukey’s Test as a post-hoc analysis was used for discrete data and parametric statistical method using an independent *t*-test was used for continuous data. For non-normally distributed data, the Kruskal-Wallis test followed by the Mann-Whitney *U* test was used for discrete data and non-parametric methods using the Mann-Whitney *U* test were used for continuous data.

## Results

### TEA tolerance test

**Fig. 2:**
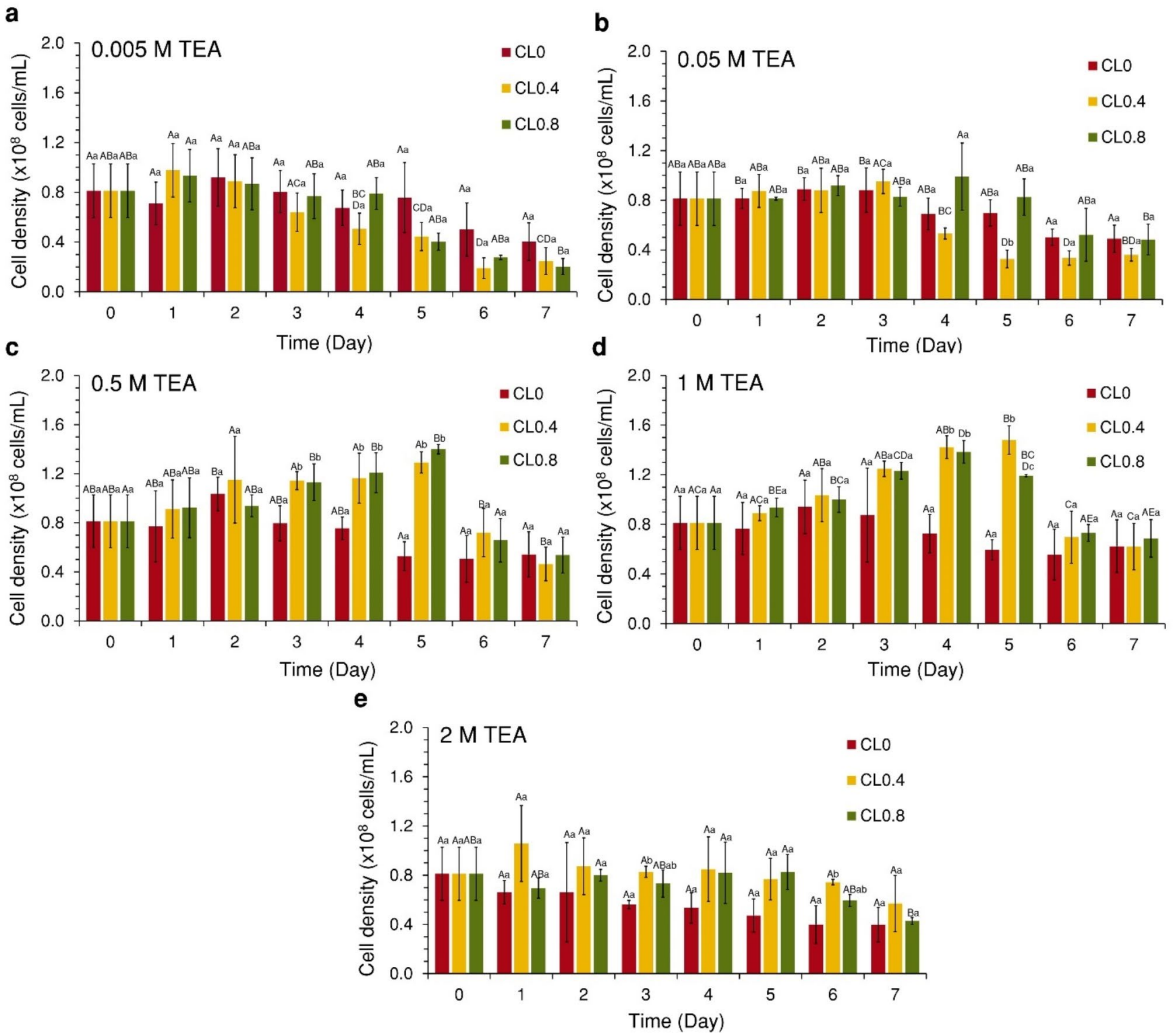
TEA tolerance test of *S. acuminatus* to TEA solution at different concentrations: (**a**) 0.005, (**b**) 0.05, (**c**) 0.5, (**d**) 1, and (**e**) 2 M, using CO_2_ loading levels of 0, 0.4, and 0.8 mol CO_2_/mol TEA, respectively, over a period of 7 days. Bar chart indicates cell density. CL = CO_2_ loading, uppercase letters represent the effect of the same CO_2_ loading levels from day 0 to day 7, and lowercase letters represent the effect of different CO_2_ loading levels on the same day. The same letters indicate that the mean values of those data sets are not statistically significantly different (*p* > 0.05) using Tukey’s post hoc analysis. (Mean $\:\pm\:$ StDev; *n* = 3).

**Fig. 3:**
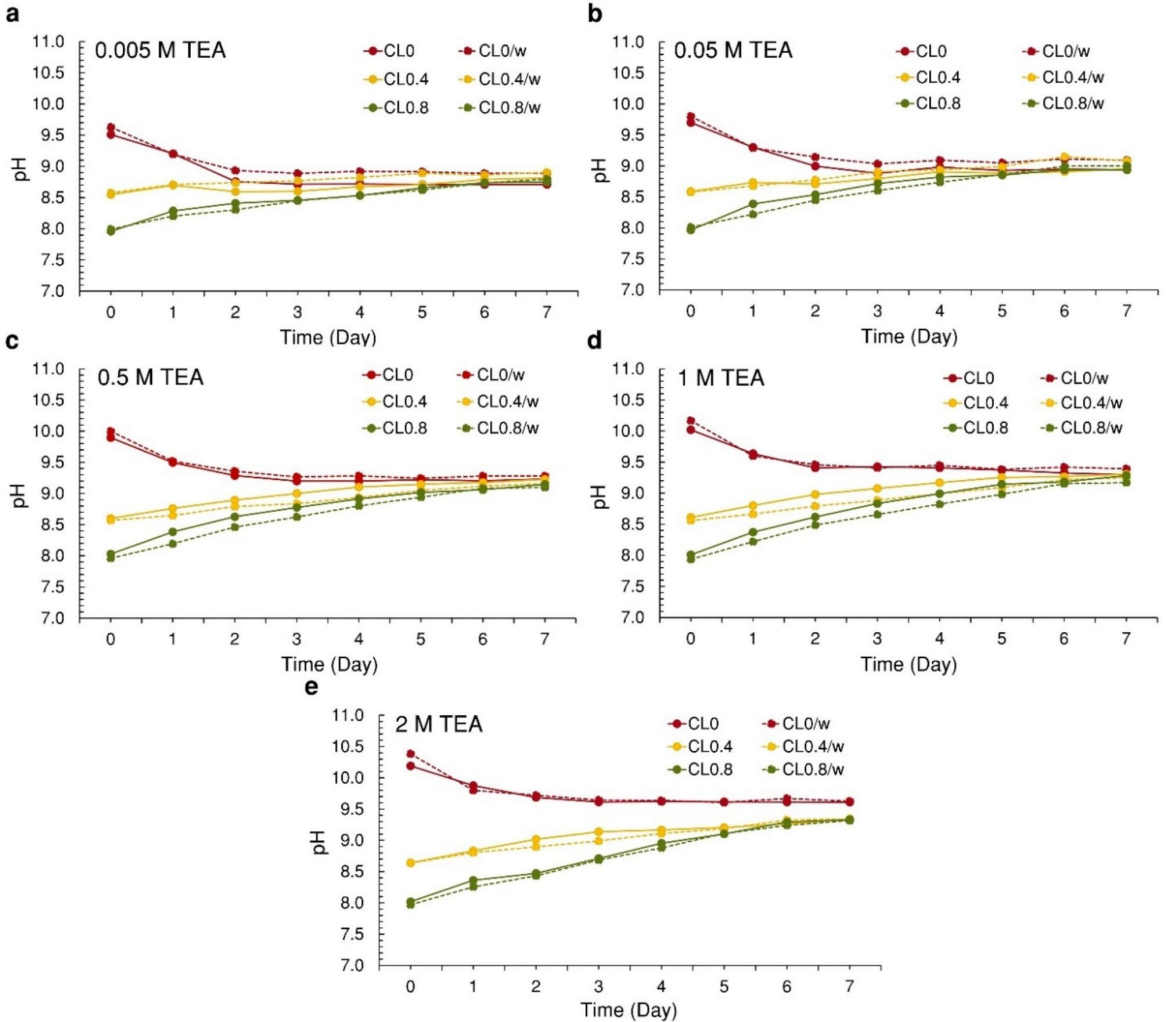
TEA tolerance test of *S. acuminatus* to TEA solution at different concentrations: (**a**) 0.005, (**b**) 0.05, (**c**) 0.5, (**d**) 1, and (**e**) 2 M, using CO_2_ loading levels of 0, 0.4, and 0.8 mol CO_2_/mol TEA, respectively, over a period of 7 days. Solid line graph indicates pH with microalgae, and the dashed line graph indicates pH without microalgae. CL = CO_2_ loading.

This study investigated the effects of varying concentrations of TEA solution and CO_2_ loading (CL) on the growth of *S. acuminatus* by measuring cell density (Fig. 2) and pH (Fig. 3) daily, then analyzed with statistical comparisons (Table 1). It was found that microalgal cell density statistically significant increased when CO_2_ was presented into the TEA solution, particularly at a TEA concentration of 1 M. The highest cell density was observed as 1.82-fold (day 5) and 1.70-fold (day 4) at CL0.4 and CL0.8, respectively. Meanwhile, cell density of 0.5 M TEA conditions increased up to 1.59-fold (day 5) and 1.72-fold (day 5) at CL4 and CL0.8, respectively, although these were not significant differences from CL0, while microalgae only grew little in 0.005, 0.05, and 2 M TEA conditions. Analysis of pH across different TEA concentrations and CL (Fig. 3) revealed a correlation with the amount of dissolved inorganic carbon (DIC) in the solution. The pH not only reflected the presence of carbon in the solution but also indicated the metabolic activity of microalgae^40^. For CL0, the initial pH ranged from 9.51 to 10.19, lower than the without microalgae control for all conditions. In this case, cell density peaked between 0 and 2 days before declining. For CL0.4 and CL0.8, the initial pH ranged from 8.55 to 8.64 and 7.96–8.02, respectively. Afterward, the pH increased to range of 8.81–9.35 and 8.75–9.33, respectively, exceeding all control conditions.

**Table 1:** Statistical analysis of the TEA tolerance test comparing CO_2_ loading levels of 0–0.4 and 0–0.8 at different TEA concentrations. The symbol (*) indicates statistically significant differences in cell density.

TEA (M)	Statistical analysis of CO_2_ loading 0 and 0.4			Statistical analysis of CO_2_ loading 0 and 0.8		
	df	t	*p*-value	df	t	*p*-value
0.005	14	0.92	> 0.05	14	0.56	> 0.05
0.05	14	0.79	> 0.05	14	0.62	> 0.05
0.5	14	2.03	> 0.05	14	1.94	> 0.05
1	14	2.30	< 0.05*	14	2.55	< 0.05*
2	14	3.57	< 0.05*	14	2.12	> 0.05

### Binder toxicity and adhesion tests

**Fig. 4:**
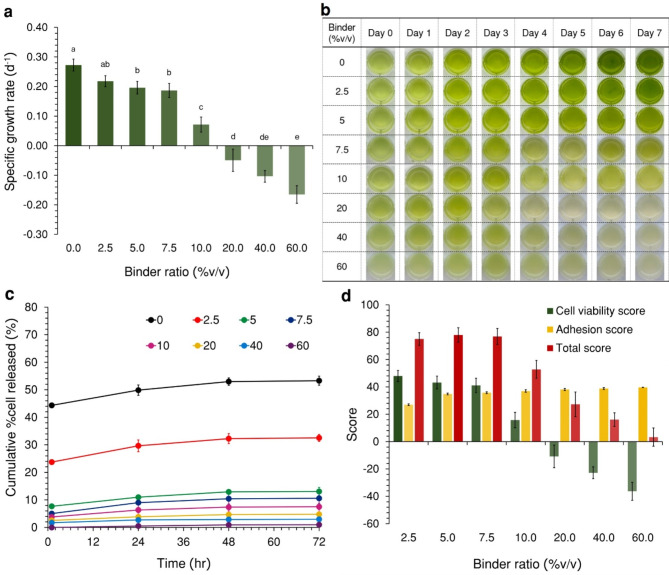
(**a**) Binder toxicity test of acrylic medium to *S. acuminatus* using specific growth rate along with (**b**) images of the test over 7 days, (**c**) binder adhesion test of cells on the biocomposites at different the binder ratios (0, 2.5, 5, 7.5, 10, 20, 40, and 60%v/v) using cumulative percentage of cell released, and (**d**) decision matrix for selecting the appropriate binder ratio; the same lower case letters indicate that the mean values of those data sets are not significantly different (*p* > 0.05) using Tukey’s post hoc analysis (Mean $\:\pm\:$ StDev; *n* = 3).

The specific growth rate through comparing cell density of *S. acuminatus* at each binder ratio (Fig. 4a) demonstrated the binder hindered the cell growth. The specific growth rate significantly decreased with increasing binder ratios (ANOVA: *F* (7,23) = 128.56, *p *< 0.05), with the specific growth rate lower than the biotic control (0.27 ± 0.02 d^−1^) at all concentrations. However, the microalgae were still able to grow at low binder concentrations (2.5–10%v/v) indicating that the cells could survive under these binder ratios. The color of cells (Fig. 4b) was lighter green with increasing binder ratio showing a reduction in the chlorophyll *a* within the cell structure. This was particularly evident at concentrations above 20%v/v, which resulted in a negative specific growth rate, confirming that *S. acuminatus* could not survive at these concentrations. For the binder adhesion test, the cumulative percentage of cells released (Fig. 4c) from the loofah after 72 h significantly decreased with increasing binder ratios (ANOVA: *F*(7,23) = 380.25, *p* < 0.05) indicating acrylic medium had the potential to increase binding affinity of cells to the loofah surface.

After achieving the binder toxicity and adhesion tests, decision matrix (Fig. 4d) was used for selecting an appropriate binder ratio to produce microalgae-loofah biocomposites. The results showed that the 5%v/v of cell/binder mixture received the highest total score because this ratio was not excessive, affecting less toxic to the microalgae while providing sufficient adhesive capability to the loofah. Therefore, this binder ratio was used to produce living microalgae-loofah biocomposites for CO_2_ removal test.

**Fig. 5:**
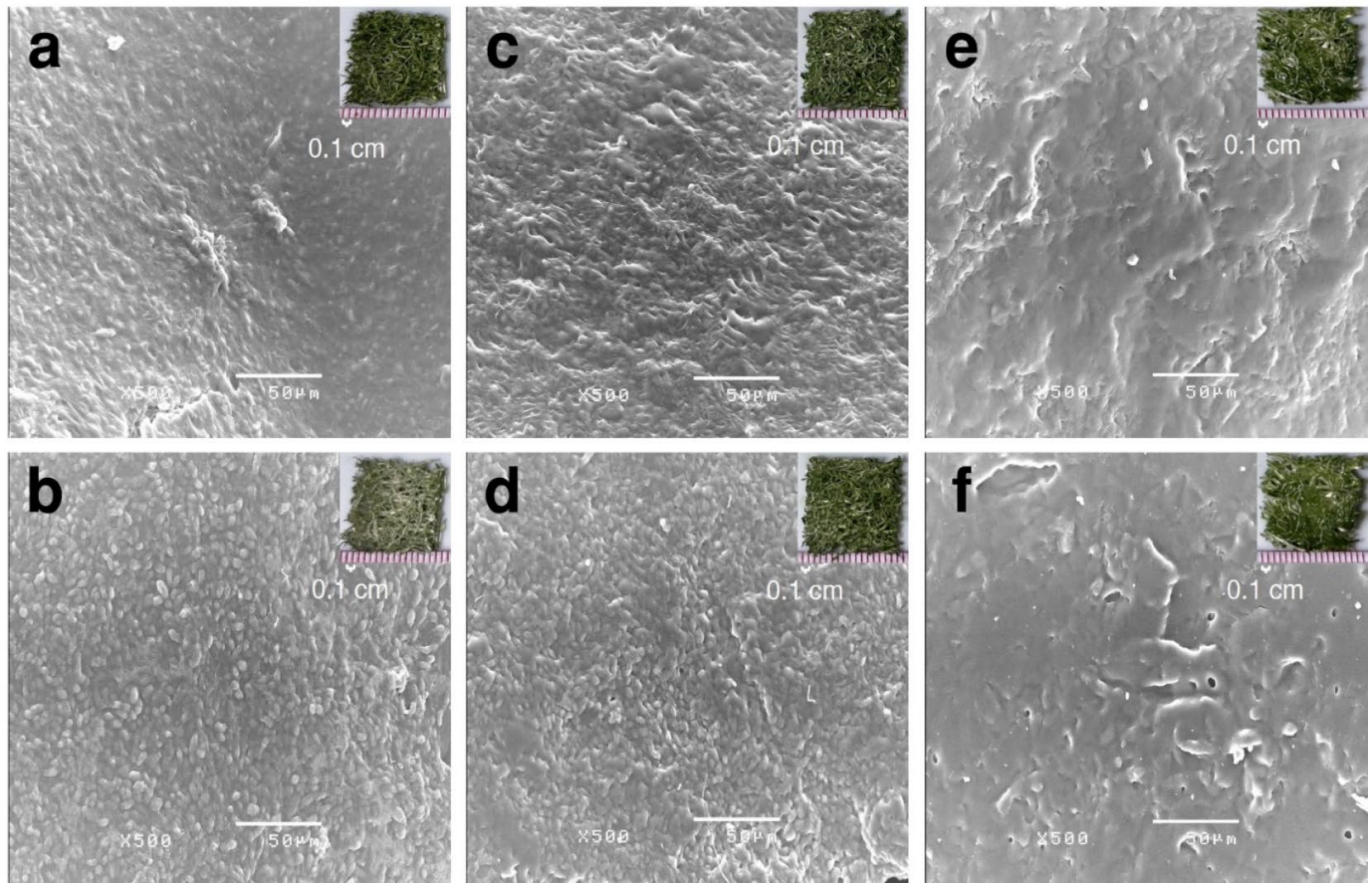
SEM images present the morphology of the microalgae-loofah biocomposites before and after the binder adhesion test at binder ratios of 0% (a and b), 5% (c and d), and 60% (e and f) %v/v, respectively, at 500$\:\times\:$ magnification.

From the SEM images (Fig. 5) of the biocomposites before and after the binder adhesion test, the cells on the loofah with no binder (0%v/v) before (Fig. 5a) the test showed that *S. acuminatus *can produce extracellular polymeric substance (EPS), acting as a natural binder. EPS can protect the cells from other microorganisms and aid in cell adhesion to surfaces^41^. The EPS layer was observed to have detached after the test (Fig. 5b), making the cells more visible. With 5%v/v of the binder before the test (Fig. 5c), it was observed that the cells were covered more tightly than no binder condition indicating that the binder covered the cells. Afterward, the binder had partially detached (Fig. 5d), making the cells visible, but the cell shapes were unclear compared to no binder condition suggesting that some binder still encapsulated the cells. Lastly, for both before (Fig. 5e) and after (Fig. 5f) the test of 60%v/v binder condition, it was observed that a dense binder layer covered the cells, making them invisible. Excessive binder coverage of the cells may impede light, reducing the photosynthesis rate and consequently the CO_2_ removal rate. The optimal binder content was more beneficial for cell survival and adhesion to the biocomposites. Therefore, 5%v/v acrylic medium was selected for CO_2_ removal test based on the decision matrix.

### Carbon dioxide removal test

**Fig. 6:**
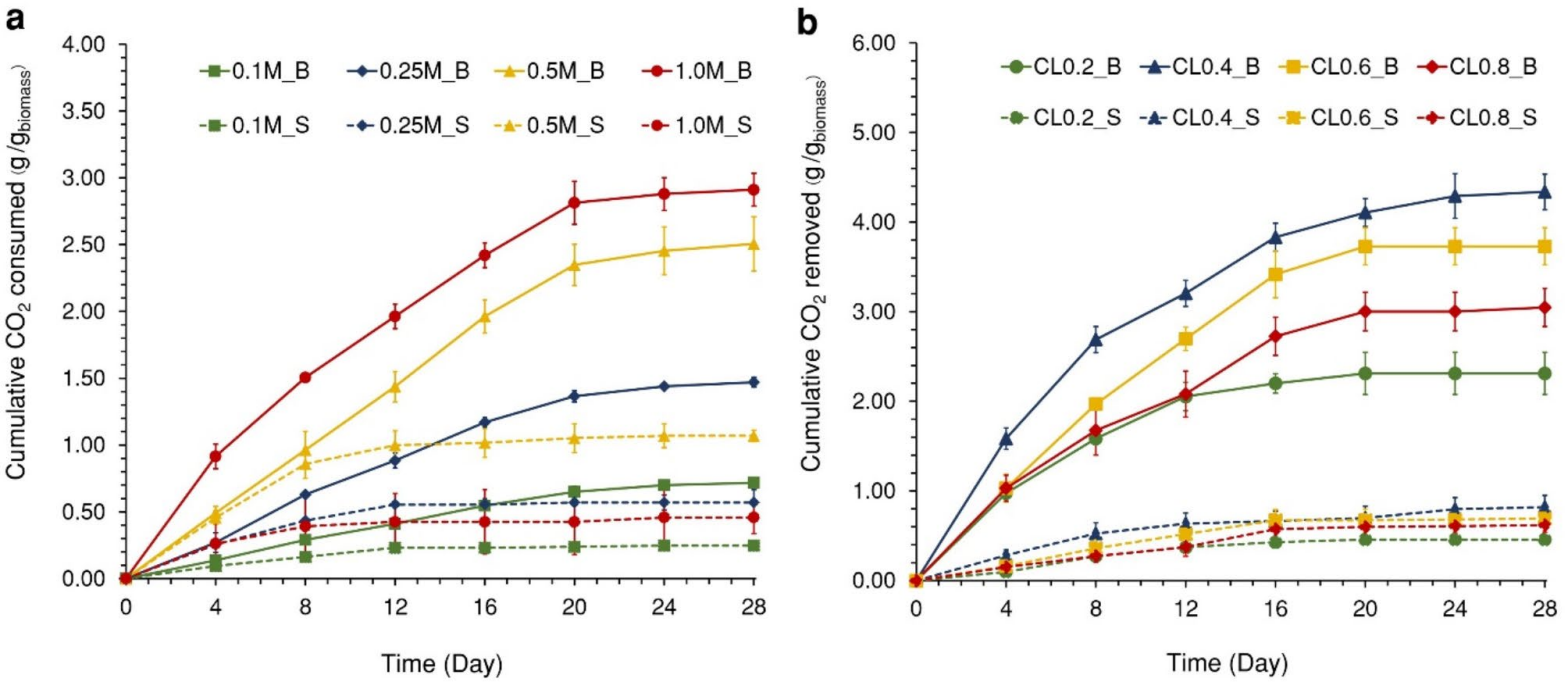
CO_2_ removal test using the biocomposites system (B) and the suspension system (S) from (**a**) TEA solution at CO_2_ loading of 0.8 mol CO_2_/mol TEA, with varying TEA solution concentrations (0.1, 0.25, 0.5, and 1 M, respectively) and (**b**) 1 M TEA solution at different CO_2_ loading levels (0.2, 0.4, 0.6, and 0.8 mol CO_2_/mol TEA, respectively), over a period of 28 days. Every 4 days, the TEA solutions were measured the amount of CO_2_ removed and the TEA solutions were refreshed into the bottle with the same concentration and CO_2_ loading. (Mean $\:\pm\:$ StDev; *n* = 3).

This assay investigated CO_2_ removal from TEA solutions at varying concentrations, including 0.1, 0.25, 0.5, and 1 M, both two cultivation systems: Biocomposites system (B) and suspended cell system (S), over a 28-day period (Fig. 6a). The results showed that the biocomposites system achieved the highest cumulative CO_2_ removal at 1.0M_B, with cumulative removal of 2.91 ± 0.12 g_CO2_/g_biomass_. In the suspended system, the highest cumulative CO_2_ removal was observed at 0.5M_S, with cumulative removal of 1.28 ± 0.04 g_CO2_/g_biomass_. The results suggested that the biocomposites system outperformed 2–4 times greater CO_2_ removal than the suspended system at the same TEA concentrations, with statistically significant differences (Table 2).

This study aimed to develop biocomposites capable of withstanding high TEA concentrations. Hence, 1.0 M TEA was selected for further testing to evaluate the effect of different CO_2_ loading (CL) levels (0.2, 0.4, 0.6, and 0.8 mol CO_2_/mol TEA) on CO_2_ removal in both cultivation systems over a 28-day period (Fig. 6b). It was found that the biocomposites system demonstrated the highest cumulative CO_2_ removal at CL levels of 0.4, 0.6, 0.8, and 0.2, observing cumulative CO_2_ removal of 4.34 ± 0.20, 3.73 ± 0.21, 3.05 ± 0.21, and 2.31 ± 0.23 g_CO2_/g_biomass_, respectively. Similarly, the suspended system showed a consistent trend, though the biocomposite system exhibited significantly higher CO_2_ removal, achieving 3–5 times greater removal at the same CL levels (Table 2). From the observations, amount of cumulative CO_2_ removal for both the biocomposites system and the suspended system follows a similar trend. This may be due to the excessive amount of HCO_3_^-^ in CL0.6_B and CL0.8_B, which became too high for the microalgae to utilize effectively, thereby reducing the CO_2 _removal rate. On the other hand, CL0.2_B had insufficient carbon sources, leading to the least utilization. Providing excessive or insufficient carbon sources could stress the microalgae, resulting in reduced growth^42^.

**Table 2:** Statistical analysis of the CO_2_ removal test on TEA concentration differences (0.1, 0.25, 0.5, and 1.0 M), and CO_2_ loading levels (0.2, 0.4, 0.6, and 0.8 mol CO_2_/mol TEA) comparing biocomposites and suspended systems over 28 days. The symbol (*) indicates statistically significant differences in the amount of CO_2_ removal.

Condition	CO_2_ removal of biocomposites system (g_CO2_/g_biomass_)	CO_2_ removal of suspended system (g_CO2_/g_biomass_)	Effect of TEA concentration		
			df	*t*	*p*-value
0.1 M	0.72 ± 0.04	0.25 ± 0.02	12	3.29	< 0.05*
0.25 M	1.47 ± 0.04	0.57 ± 0.04	12	2.98	< 0.05*
0.5 M	2.51 ± 0.20	1.28 ± 0.04	12	2.59	< 0.05*
1.0 M	2.91 ± 0.12	0.46 ± 0.12	12	6.12	< 0.05*
CL0.2	2.31 ± 0.23	0.46 ± 0.05	12	8.06	< 0.05*
CL0.4	4.34 ± 0.20	0.82 ± 0.13	12	7.19	< 0.05*
CL0.6	3.73 ± 0.21	0.69 ± 0.09	12	5.80	< 0.05*
CL0.8	3.05 ± 0.21	0.62 ± 0.08	12	6.22	< 0.05*

## Discussion

This research aimed to explore the feasibility of CO_2_ removal from CO_2_-rich TEA solution using microalgae-loofah biocomposites. The process of CO_2_ removal can be divided into chemical (before entering microalgal cells) and biological (inside the cells) aspects. Chemically, TEA can react with water and CO_2_, undergoing hydrolysis to form protonated TEA (TEAH^+^) and HCO_3_^−^. HCO_3_^−^ serves as a crucial nutrient source for microalgae instead of supplying CO_2 _gas^14^. HCO_3_^−^ can be transported into the cell and converted to CO_2_ using the CO_2 _concentrating mechanism (CCM)^43,44^. Especially for the biocomposite systems, Caldwell et al. (2021)^34^ outlined the CO_2_ capture mechanism in the biocomposites as follows: The process begins in the liquid phase, which consists of the culture medium and, in our cases, CO_2_ rich-TEA or HCO_3_^−^. These compounds are then absorbed into the loofah, representing the solid phase. The loofah’s hydrophilic properties facilitate the retention of nutrients and moisture, generating capillary forces through its multilayered fibers to aid nutrient transport. The compounds in the liquid phase pass through the porous layer of the binder to reach the microalgae. Biologically, the CO_2_ removal mechanisms in biocomposite systems are like those in suspended systems. Microalgae can assimilate dissolved inorganic carbon (DIC) in the form of HCO_3_^−^ pools, through the CCM, then stored HCO_3_^−^ is converted into CO_2 _by carbonic anhydrase near the active center of ribulose-1,5-bisphosphate carboxylase/oxygenase (RuBisCO), at the pyrenoids region within the chloroplasts for further Calvin-Benson cycle^45^. In the Calvin-Benson cycle, CO_2 _reacts with ribulose-1,5-bisphosphate (RuBP) under the catalysis of RuBisCO, using ATP and NADPH generated during the light-dependent reactions of photosynthesis. These reactions form glyceraldehyde-3-phosphate (G3P), with five molecules regenerated into RuBP and one molecule diverted to other metabolic pathways for storage as various biomolecules^25,45^.

The pH of the TEA solution with CO_2_ loading increased across all concentrations due to the microalgal cells utilizing HCO_3_^−^. During this carbon species conversion process, hydroxide ions (OH^−^) could be released outside the cell. These OH^−^ could react with TEAH^+ ^to regenerate TEA, thereby raising the pH of the system^15^. This can be observed as the pH of the TEA solution with microalgae being higher than the control conditions (TEA without microalgae). The cell density increases with the TEA concentration, becoming more noticeable at concentrations of 0.5 and 1 M with CO_2_ loading. The survival of microalgae may be due to the TEA concentration increases, meaning the HCO_3_^−^ concentration in the solution increases accordingly. Theoretically, 1 mol of HCO_3_^− ^equals 1 mol of TEA^15^, providing sufficient carbon sources for the microalgae. This is evident in TEA solution concentrations of 0.5 M (Fig. 2c) and 1 M (Fig. 2d), where the microalgae cells could survive up to 5 days before cell density decreased. In contrast, TEA solution concentrations of 0.005 M (Fig. 2a) and 0.05 M (Fig. 2b) have HCO_3_^−^ less than 10–200 times, possibly insufficient for growth, resulting in lower cell density and survival for only up to 2–4 days. This research used an initial density of 10^9 ^cells/mL (suitable for biocomposites fabrication^32^), necessitating higher carbon sources. The decreasing in HCO_3_^−^ could be inferred from the pH of CL0.4 and CL0.8 approaching that of CL0 (Fig. 3), with TEA solution concentrations of 0.005 M (2 days), 0.05 M (3 days), 0.5 M (5 days), and 1 M (5 days). Finally, at a TEA solution concentration of 2 M (Fig. 2e), microalgal cells are likely unable to survive in any CO_2_ loading due to various factors, such as excessive alkalinity, high viscosity limiting cell movement, or excessive HCO_3_^−^ concentration^42^. Another reason for survival of microalgae may be due to the rich CO_2_-TEA having weak alkalinity (approximately pH 8–8.5 on the first day), which prevent pH fluctuation (from approximately pH 9–10)^18^. However, CO_2 _may escape from the TEA solution into the atmosphere over time, and hydrolysis may revert it to its original TEA form, increasing the alkalinity of the solution, which could be toxic to the microalgae cells^18^. Furthermore, the effect of TEA and CO_2_ loading on inhibiting growth of microalgal cells after HCO_3_^−^ utilization remains unclear. Additional microbiological studies are necessary to investigate cellular response mechanisms in microalgae cultivation with TEA solution.

Natural loofah was selected as the solid support material for the living microalgae biocomposites due to its high surface area and void space. In-na et al. (2020)^31^ characterized the loofah structure by embedding it in a mold, dyeing the fibers black, and capturing high-resolution digital images. These images were then analyzed, which provided surface area of 950 m^2^/m^3^and a void space of up to 80%, suitable for cell immobilization and promoting light penetration^31^. Additionally, the hydrophilic properties of loofah fibers allow them to retain moisture within their structure^46^, supporting nutrient transport through capillary force^47^. The loofah also exhibits good mechanical properties, resistant to temperature and pH variations^48^. The 5%v/v of acrylic medium was selected for immobilizing microalgal cells on loofah because of its low toxicity to cells and its potential to help cells adhere to the biocomposites. Furthermore, it can form a thin and transparent film after drying, which covers the cells and allows good light penetration^31^. The binder layer may help to compromise instantaneous change from high CO_2_ concentration and alkalinity of the TEA solution. In contrast, suspended cells were directly immersed in the TEA solution, constantly directly exposed to high CO_2 _concentration and lacking proper agitation, making it harder for light to reach the cells due to the need to pass through the water layer first^49^. As a result, this leads to reducing in photosynthesis efficiency and lower CO_2_ removal rates of the suspended system compared to the biocomposites system. To ensure consistency between the biocomposites and suspended systems, initial biomass and TEA solution volumes were equalized. Effective mixing in practical culture systems is typically achieved with mechanical agitation using paddles or pneumatic systems with air injection. Mixing may help microalgae access light, nutrients, and HCO_3_^−^, potentially enhancing CO_2 _removal^50^. However, for amine-based absorption process combined with microalgae, high aeration rate should be avoided as it affects the absorbed CO_2_ in the TEA solution, and paddles may harm microalgae due to shear forces. Moreover, agitation may also affect the binder on the biocomposites over time.

Improvement in CO_2_ removal from TEA solution may increase the number of cells on loofah, but it should not block light penetration and prevent cell detachment. Additional factors may involve biocomposites fabrication process (binder type, immersion time, drying time, and drying temperature). The pH fluctuations on overall efficiency of CO_2_ removal can be addressed by carefully monitoring in real-time using probes and sensors integrated into automated systems to track changes in CL, pH, and available nutrients, which are interdependent. As microalgae remove CO_2_, the pH will increase, indicating a reduction in CL. The lean-CL TEA can then be removed and replaced with freshly rich-CL TEA, which is known as a fed-batch system. This strategy may not only mitigate pH fluctuations but also replenish the carbon source for the microalgae.

For up-scaling from laboratory to industrial scales, pilot studies are necessary to test and enhance system performance under realistic conditions, verifying the biocomposites tolerance to TEA solution in outdoor environments. Outdoor environment poses various challenges due to unpredictable weather, which can lead to variability in microalgal growth. Key factors affecting CO_2_ removal, such as light intensity and temperature, fluctuate with seasonal changes. To address these challenges, integrating effective photobioreactors (PBRs) design plays a crucial role in maintaining optimal light distribution, temperature regulation, mixing efficiency, and mass transfer, ultimately enhancing CO_2 _sequestration. Certain PBR configurations, such as bubble-column, air-lift, and stirred-tank PBRs, often encounter regions of low illumination. Alternative designs, like loop PBRs, can offer a larger surface area while maintaining efficient mixing without the need for impeller^51^. The PBR should be a closed system and optimize flow rate to prevent the escape of TEA solution and CO_2_ while ensuring compatibility with loofah fiber and the specific geographical conditions of the deployment site.

To optimize light penetration, the reactor should not be excessively large, as overlapping loofah fibers may obstruct light transmission. It is anticipated that the light intensity and quality can vary seasonally, unlike controlled laboratory conditions, necessitating adjustments in culture system thickness and reactor material selection based on geographic location. In addition, during low-light seasons, artificial lighting systems could be incorporated to maintain CO_2_ removal efficiency, alongside a temperature control system to stabilize environmental conditions. Seasonal changes also cause temperature fluctuations, requiring system design to control temperature and selecting microalgae strains adaptable to local conditions. Therefore, an automated control system should include light, temperature, CO_2_ and O_2 _levels (to prevent photorespiration). The next factor is nutrient consumption by microalgae. In laboratory settings, BG-11 medium was used, known for supporting various algae species due to its sufficient nutrients^52^. An alternative irrigation strategy can involve a spray system, which evenly distributes the TEA solution as well as BG-11 medium over the biocomposites. Other factors, screening microbial strains, and incorporating genetic engineering to enhance microalgal performance, should be explored to maximize the potential of biocomposites system. Khandelwal et al. (2021)^53^ demonstrated the performance of *Halomonas stevensii* cultivation within 2 L bio-reactor for CO_2_ bio-fixation incorporate with domestic wastewater treatment and found that with high CO_2_ fixation rate up to 4.04 g/L/d^53^. This study paves the idea for further advanced immobilizing this bacterial strain onto biocomposites, enabling simultaneous CO_2_ removal and nutrient cost reduction by utilizing inorganic compounds of wastewater.

Future research should focus on TEA recycling for the subsequent cycles of CO_2_ capture testing after regeneration by microalgae. Additionally, the production of added-valuable products, especially converting *S. acuminatus* lipids to biodiesel or other bio-oil products, maximizing CO_2_ utilization, or testing amine that can react with CO_2_ to produce HCO_3_^−^such as AMP^26 ^and methyl diethanolamine (MDEA)^54^, providing more options for integrating amine-based absorption combined with microalgae cultivation.

## Data Availability

The datasets used and/or analysed during the current study available from the corresponding author on reasonable request.
